# Supplementary figures and images for: An actin-related protein that is most highly expressed in Drosophila testes is critical for embryonic development
Source: eLife. 2021 Jul 20;10:e71279. doi: 10.7554/eLife.71279 (PMC8291977; doi:10.7554/eLife.71279)

**B**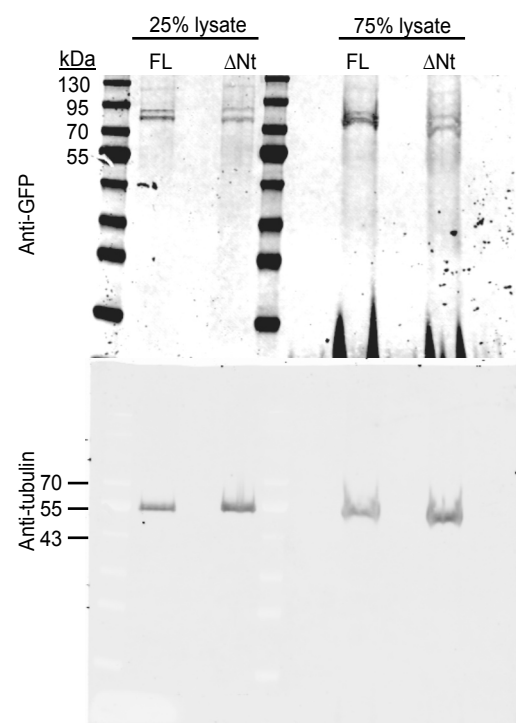**E**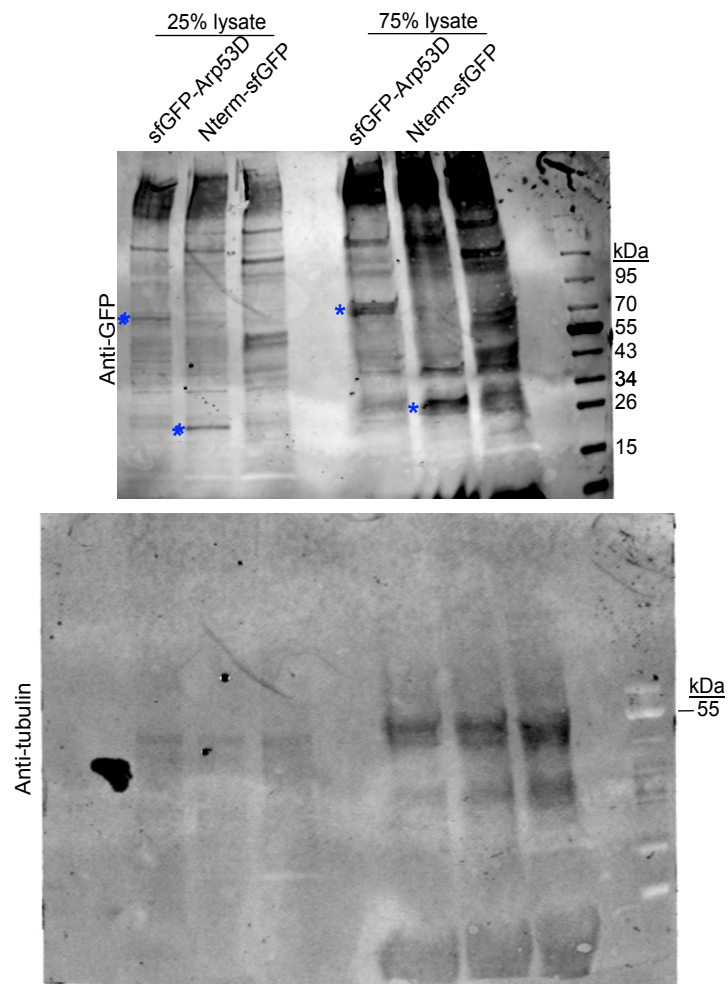

**Figure 4-figure supplement 1-source data 1**

Supplement: Figure 4—figure supplement 1—source data 1. [file elife-71279-fig4-figsupp1-data1.pdf]

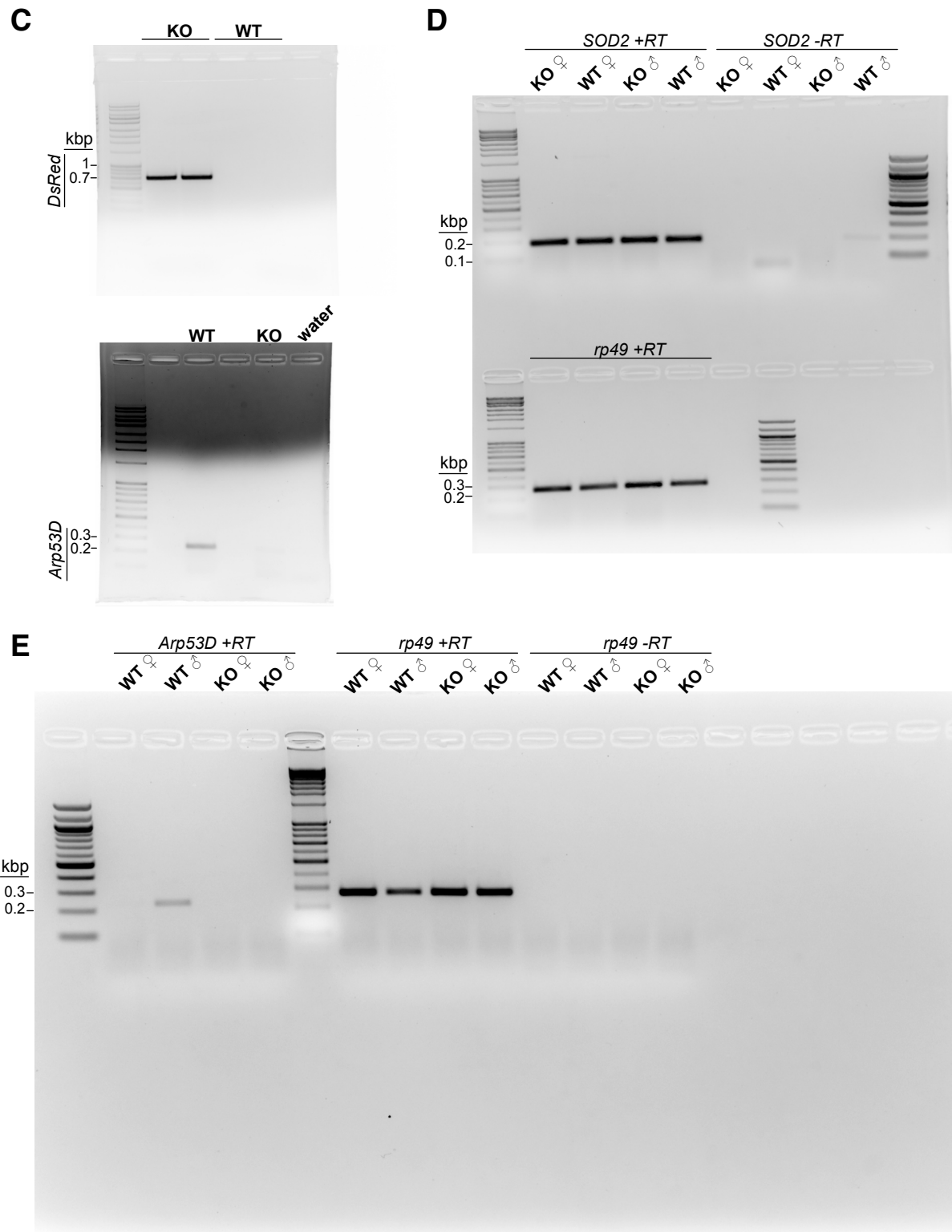

Figure 5-figure supplement 1-source data 1

Supplement: Figure 5—figure supplement 1—source data 1. [file elife-71279-fig5-figsupp1-data1.pdf]

**F**

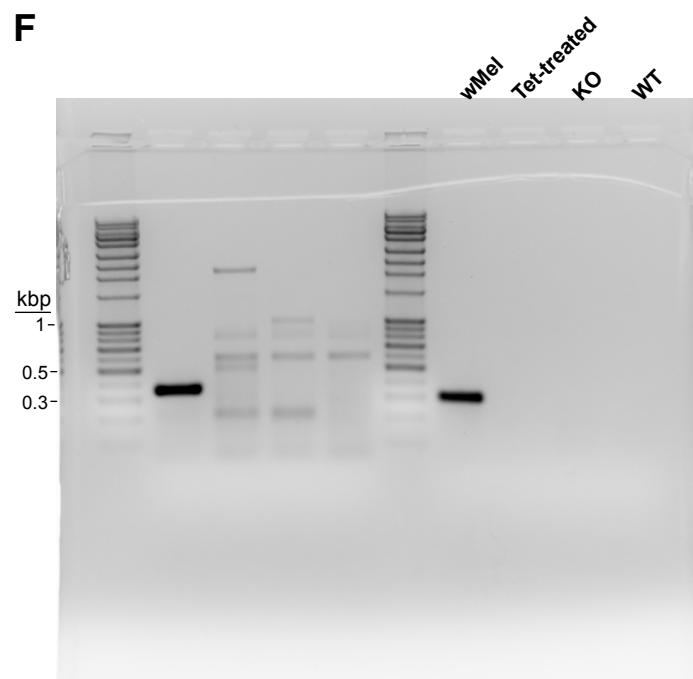

**Figure 5-figure supplement 1-source data 2**

Supplement: Figure 5—figure supplement 1—source data 2. [file elife-71279-fig5-figsupp1-data2.pdf]

**H**

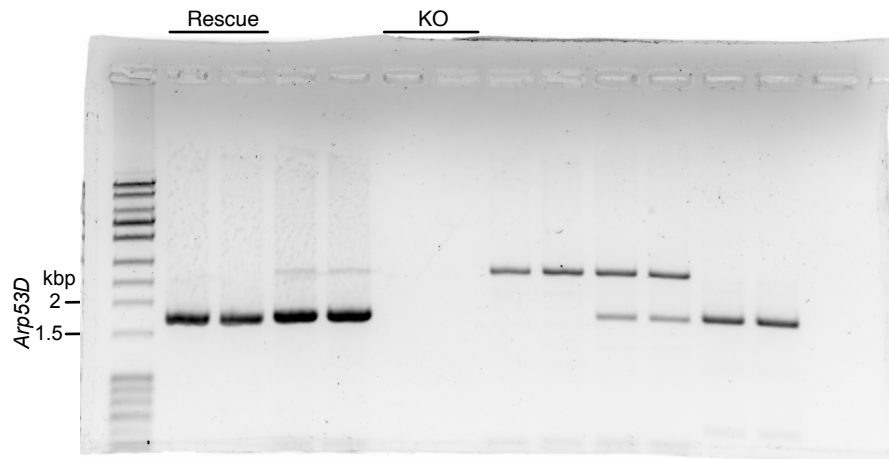

**I**

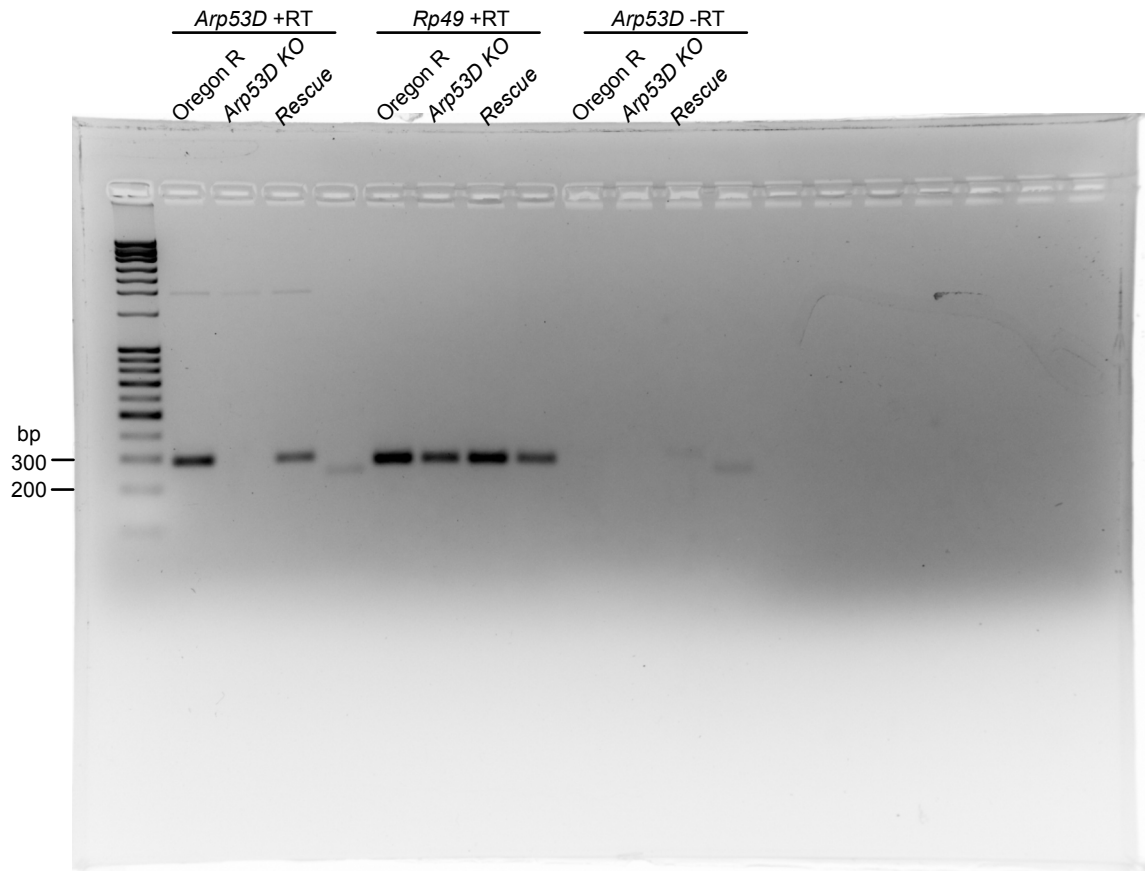

**Figure 5-figure supplement 2-source data 2**

Supplement: Figure 5—figure supplement 2—source data 2. [file elife-71279-fig5-figsupp2-data2.pdf]

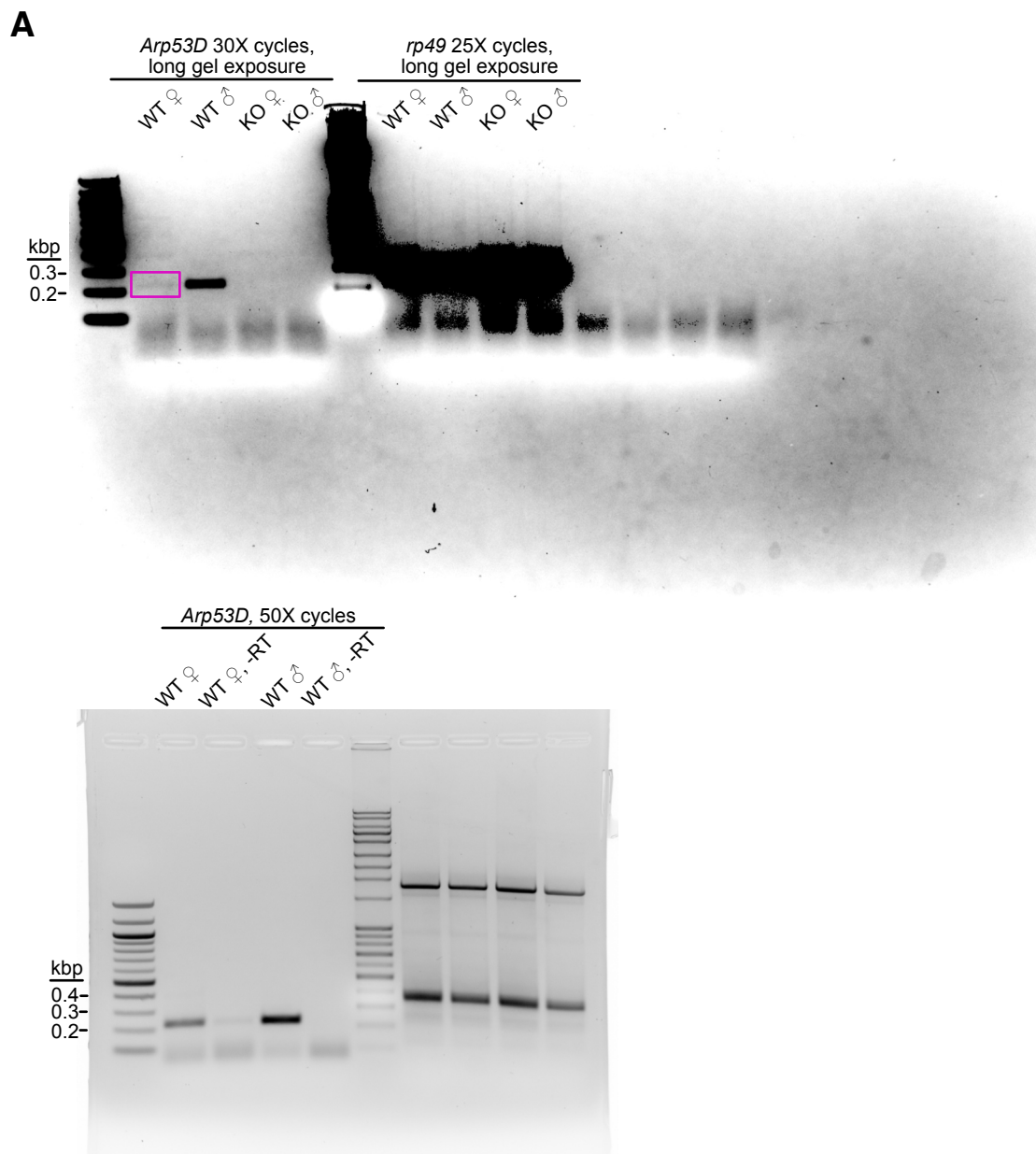

**Figure 7-figure supplement 1-source data 1**

Supplement: Figure 7—figure supplement 1—source data 1. — The uncropped gel image corresponding to the rp49 control can be found in Figure 5—figure supplement 1—source data 1. [file elife-71279-fig7-figsupp1-data1.pdf]

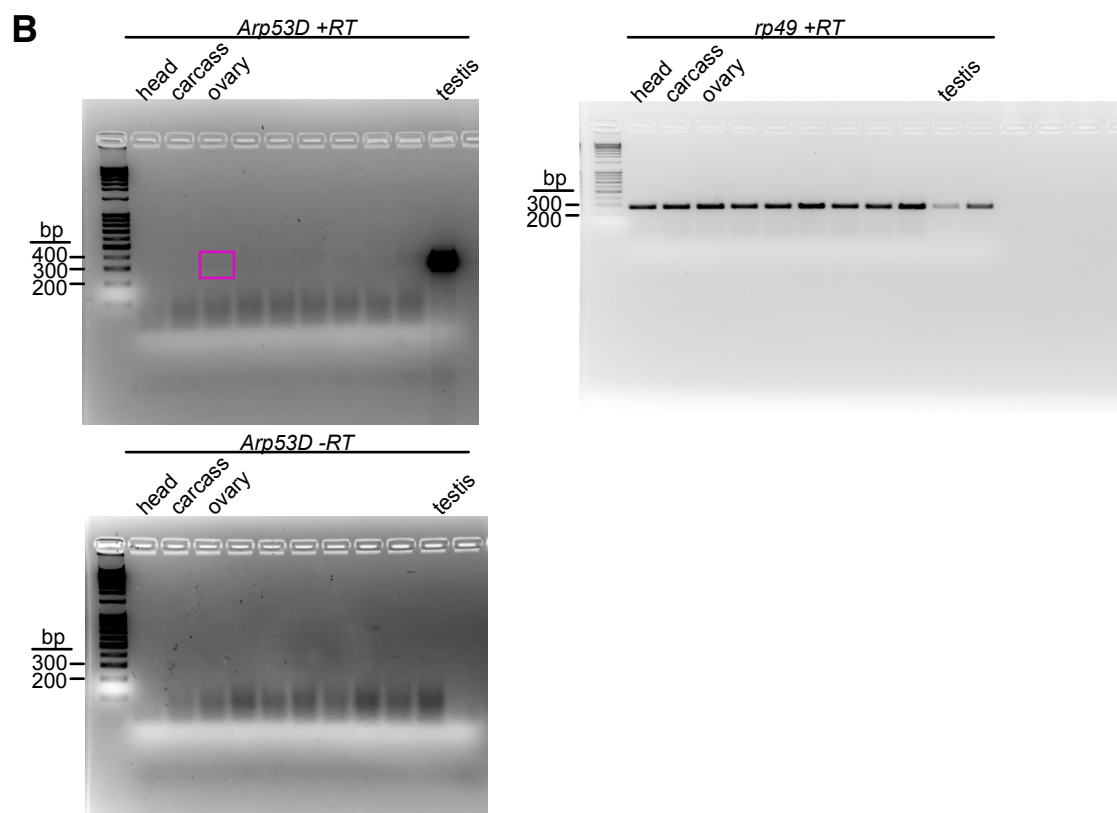

**Figure 7-figure supplement 1-source data 2**

Supplement: Figure 7—figure supplement 1—source data 2. [file elife-71279-fig7-figsupp1-data2.pdf]

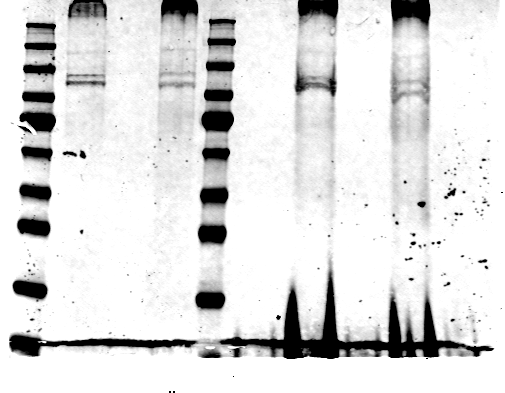

Supplement: Source data 1. [file elife-71279-data1.zip › raw source data images/Figure 4-figure supplement 1-source data 1-panel B_anti_gfp_western blot.tif]

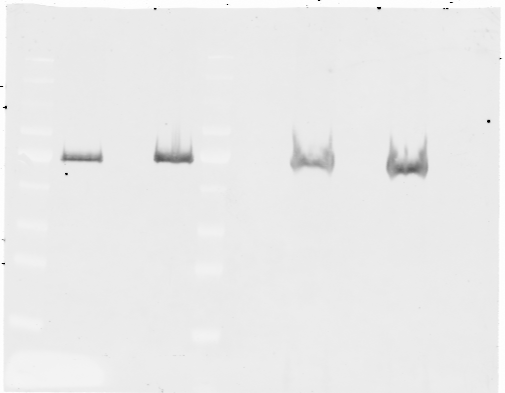

Supplement: Source data 1. [file elife-71279-data1.zip › raw source data images/Figure 4-figure supplement 1-source data 1-panel B_anti_tubulin_western blot.tif]

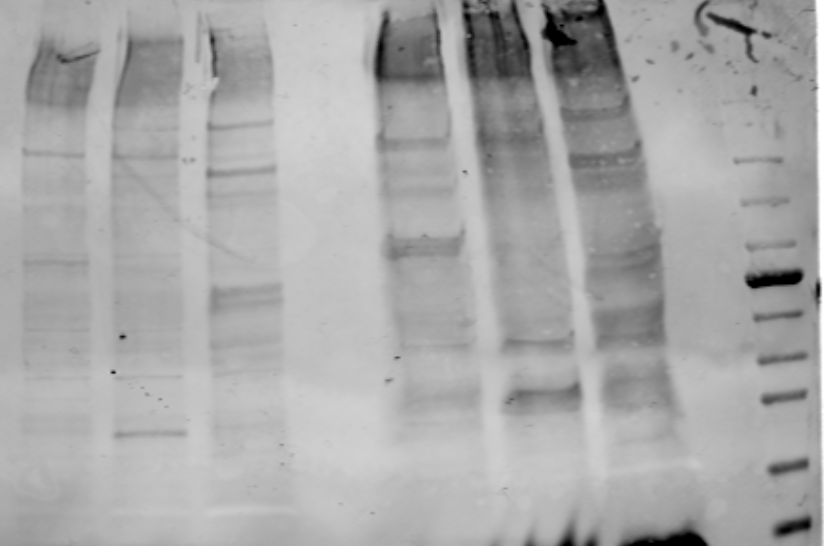

Supplement: Source data 1. [file elife-71279-data1.zip › raw source data images/Figure 4-figure supplement 1-source data 1-panel E_anti_gfp_western blot.tif]

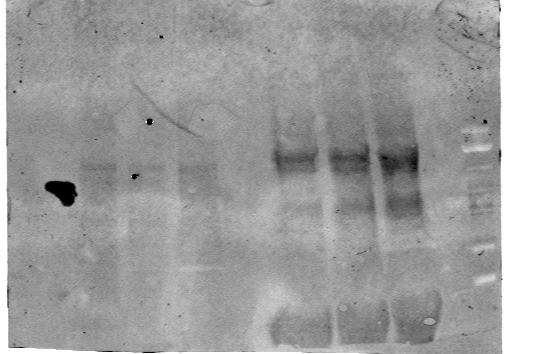

Supplement: Source data 1. [file elife-71279-data1.zip › raw source data images/Figure 4-figure supplement 1-source data 1-panel E_anti_tubulin_western blot.tif]

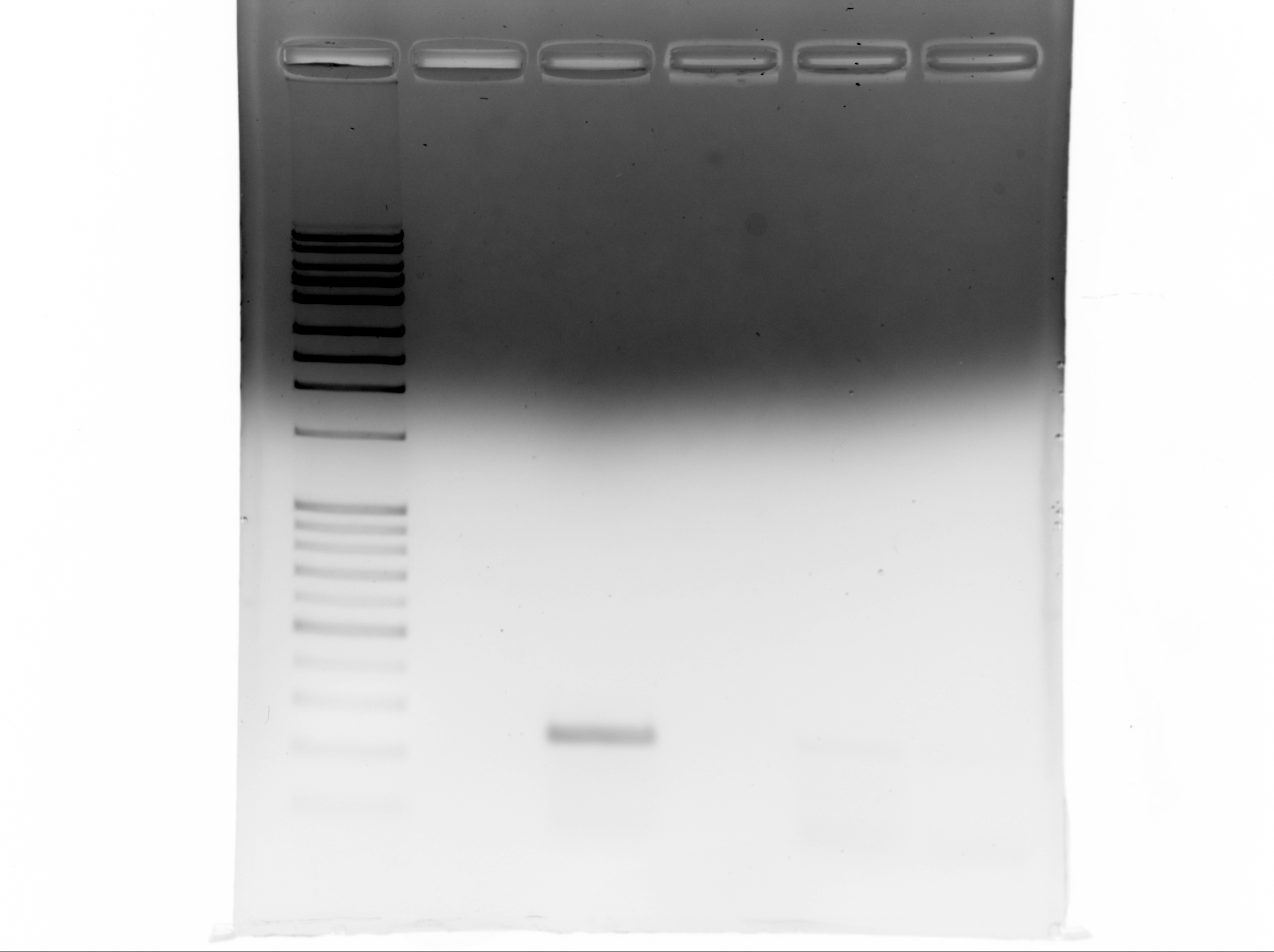

Supplement: Source data 1. [file elife-71279-data1.zip › raw source data images/Figure 5-Figure supplement 1-source data 1-panel C_Arp53D PCR.tif]

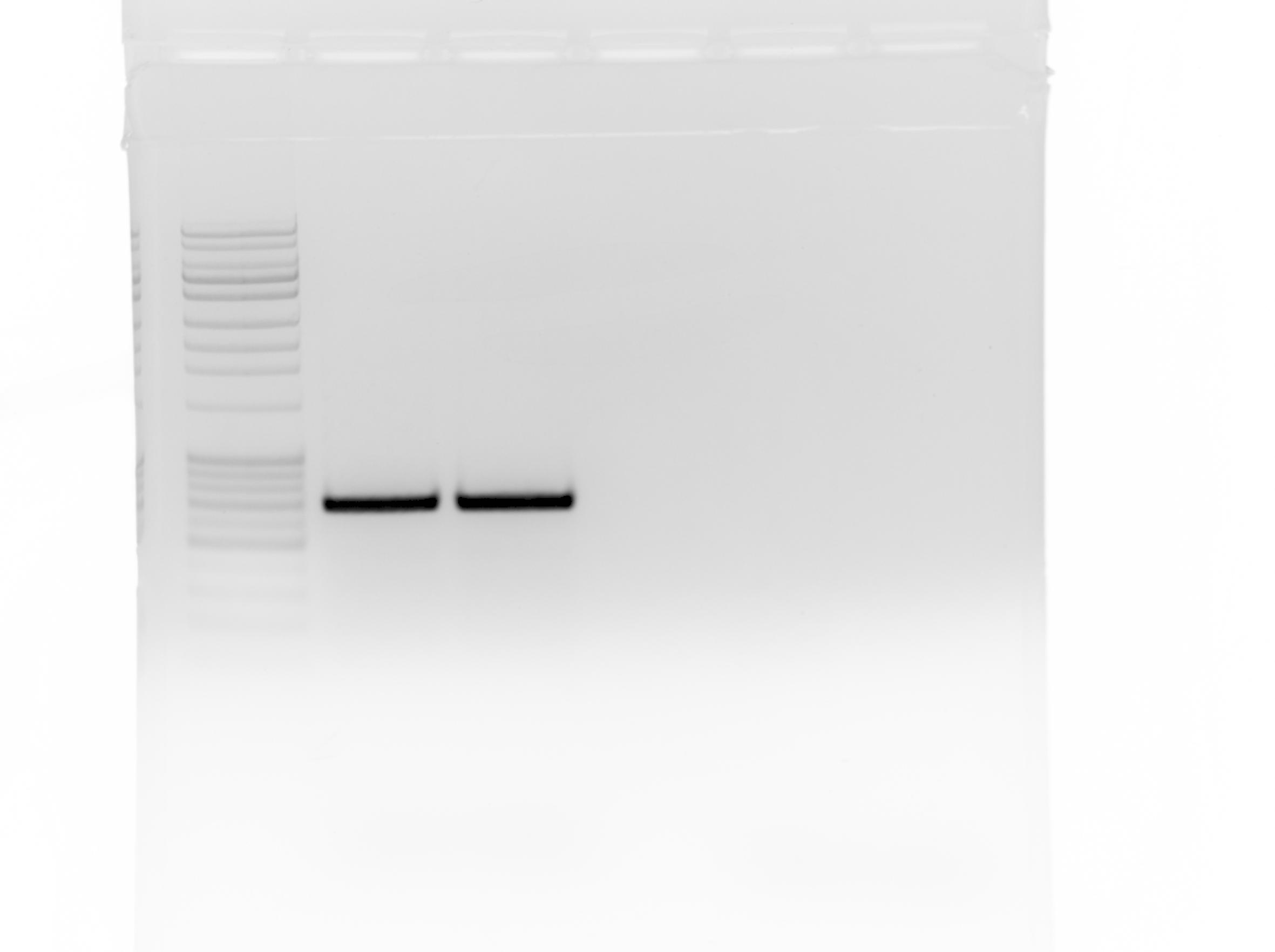

Supplement: Source data 1. [file elife-71279-data1.zip › raw source data images/Figure 5-figure supplement 1-source data 1-panel C_KO locus PCR.tif]

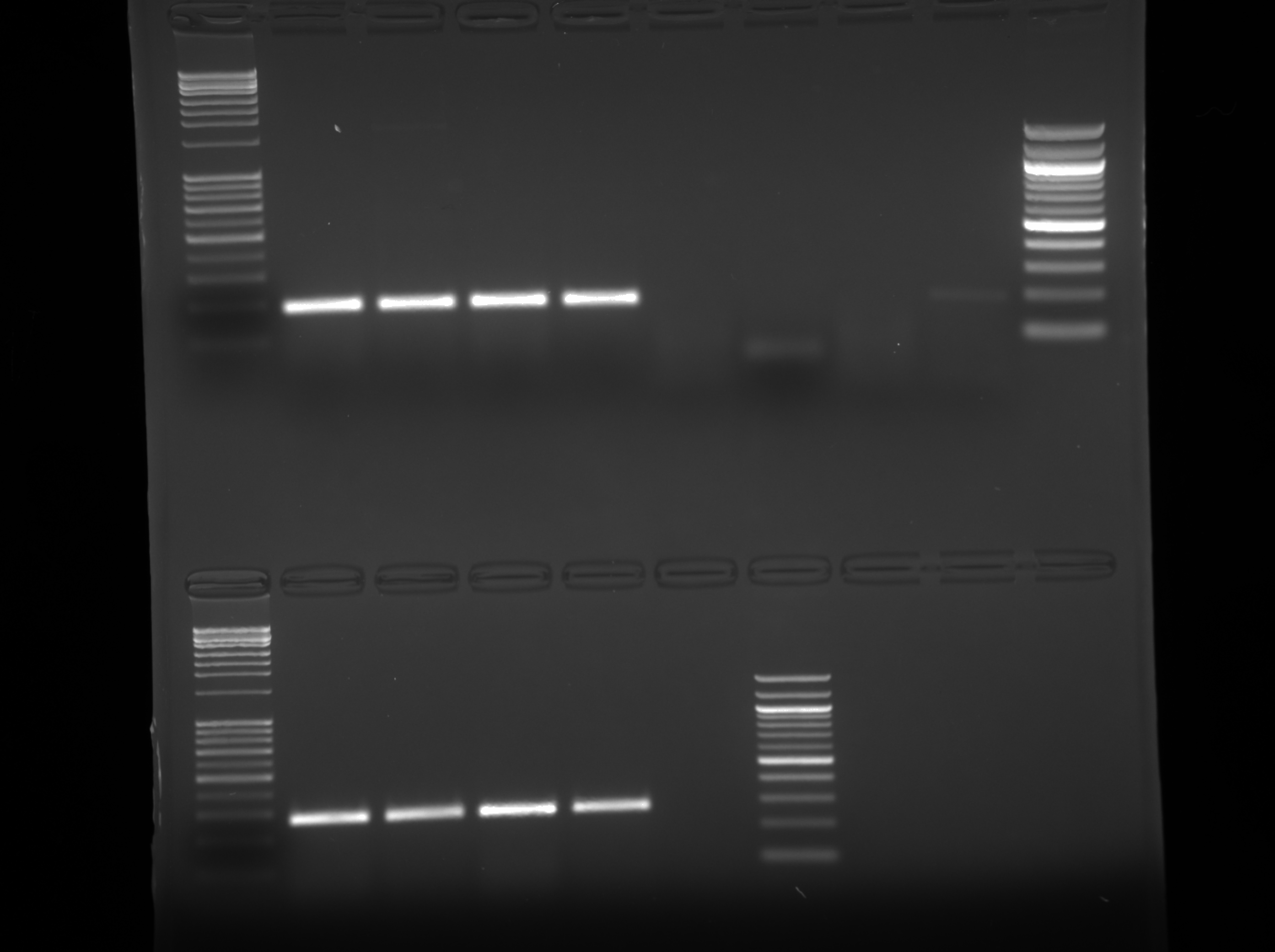

Supplement: Source data 1. [file elife-71279-data1.zip › raw source data images/Figure 5-figure supplement 1-source data 1-panel D_SOD2 expression.tif]

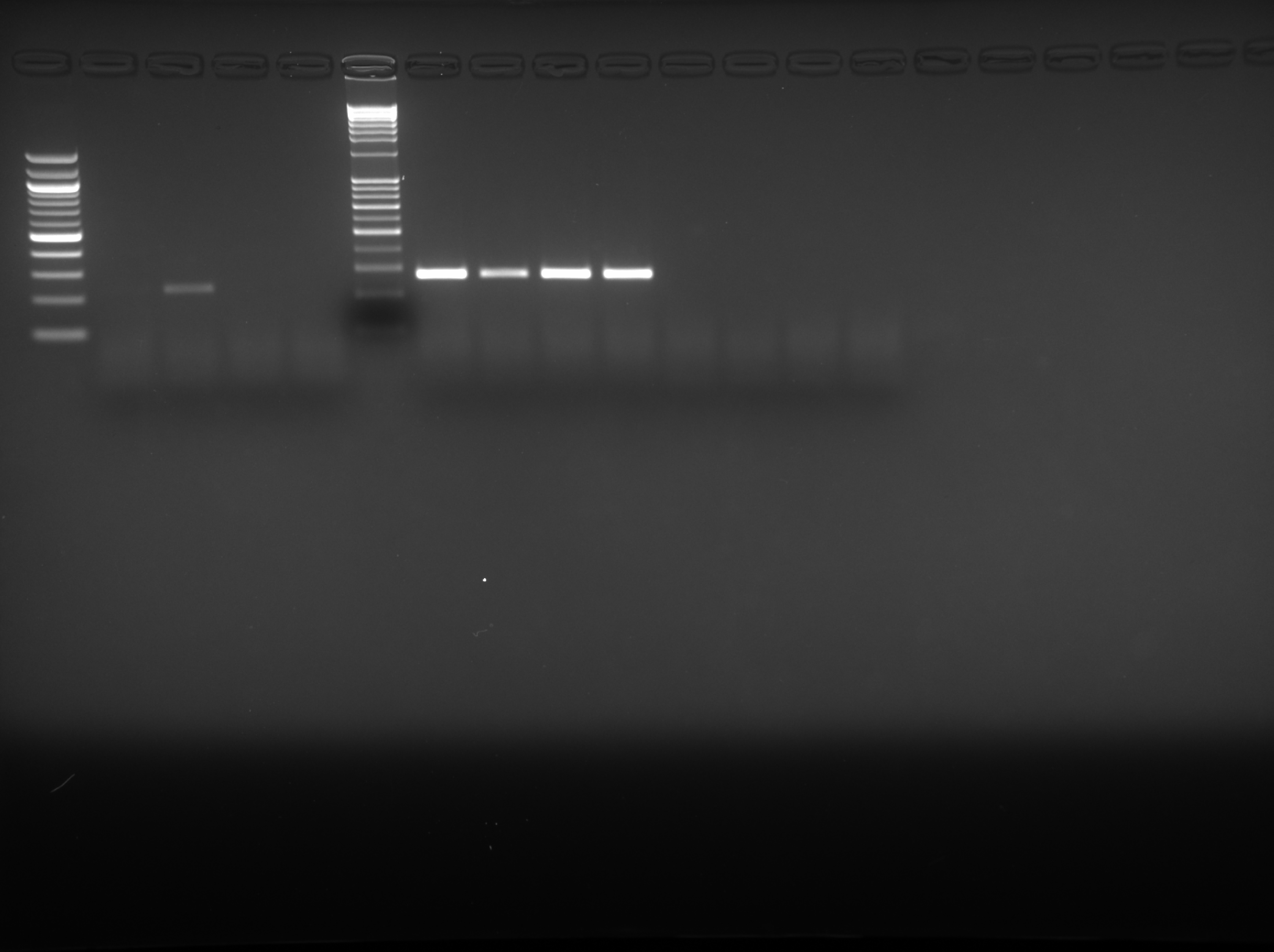

Supplement: Source data 1. [file elife-71279-data1.zip › raw source data images/Figure 5-figure supplement 1-source data 1-panel E_Arp53D expression in WT vs KO.tif]

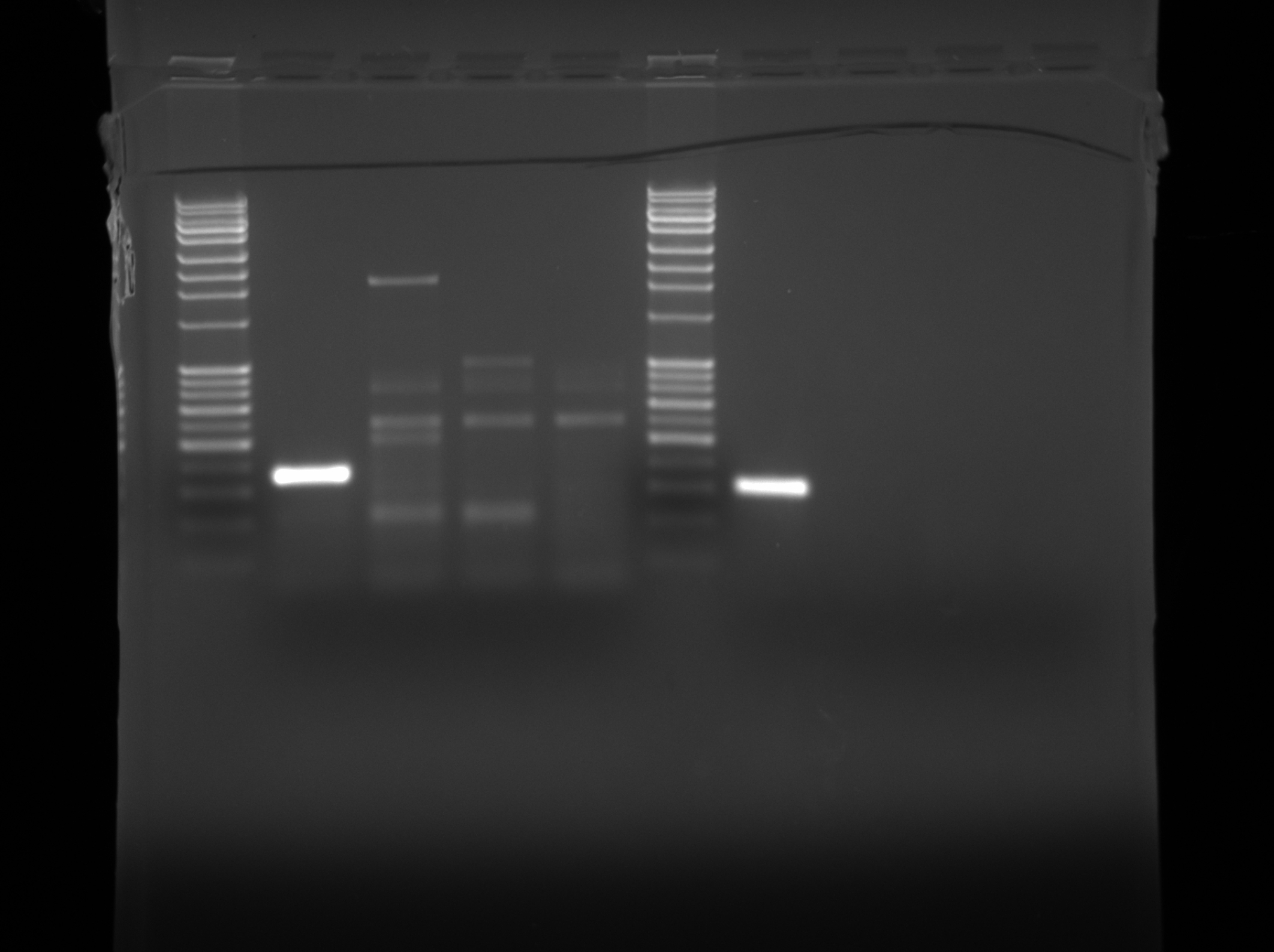

Supplement: Source data 1. [file elife-71279-data1.zip › raw source data images/Figure 5-Figure supplement 1-source data 2-panel F_Wolbachia diagnostic PCR.tif]

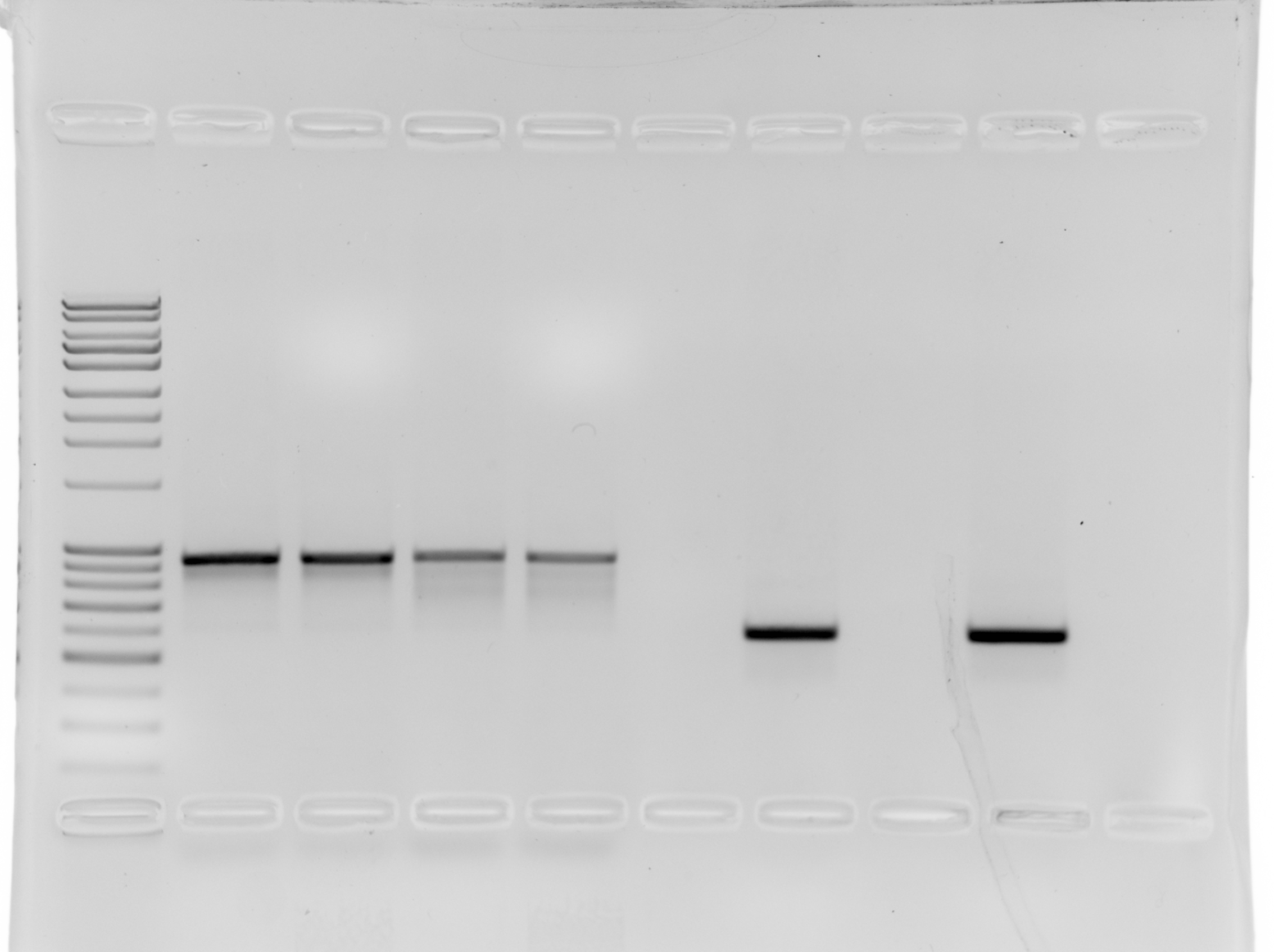

Supplement: Source data 1. [file elife-71279-data1.zip › raw source data images/Figure 5-figure supplement 2-source data 1-panel B_RNAi line validation.tif]

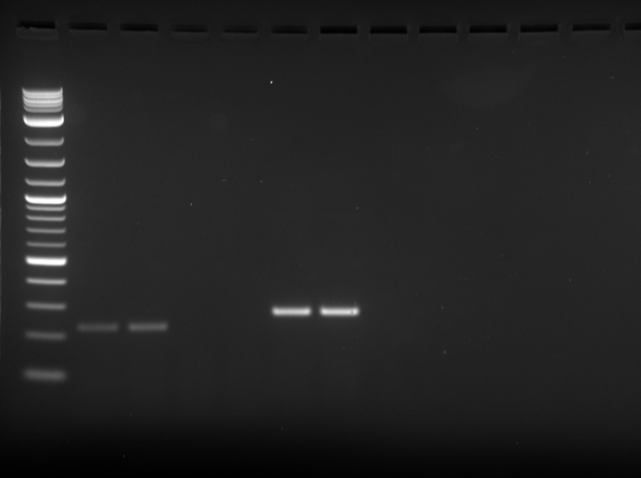

Supplement: Source data 1. [file elife-71279-data1.zip › raw source data images/Figure 5-figure supplement 2-source data 1-panel C_Arp53D expression upon knockdown.TIFF]

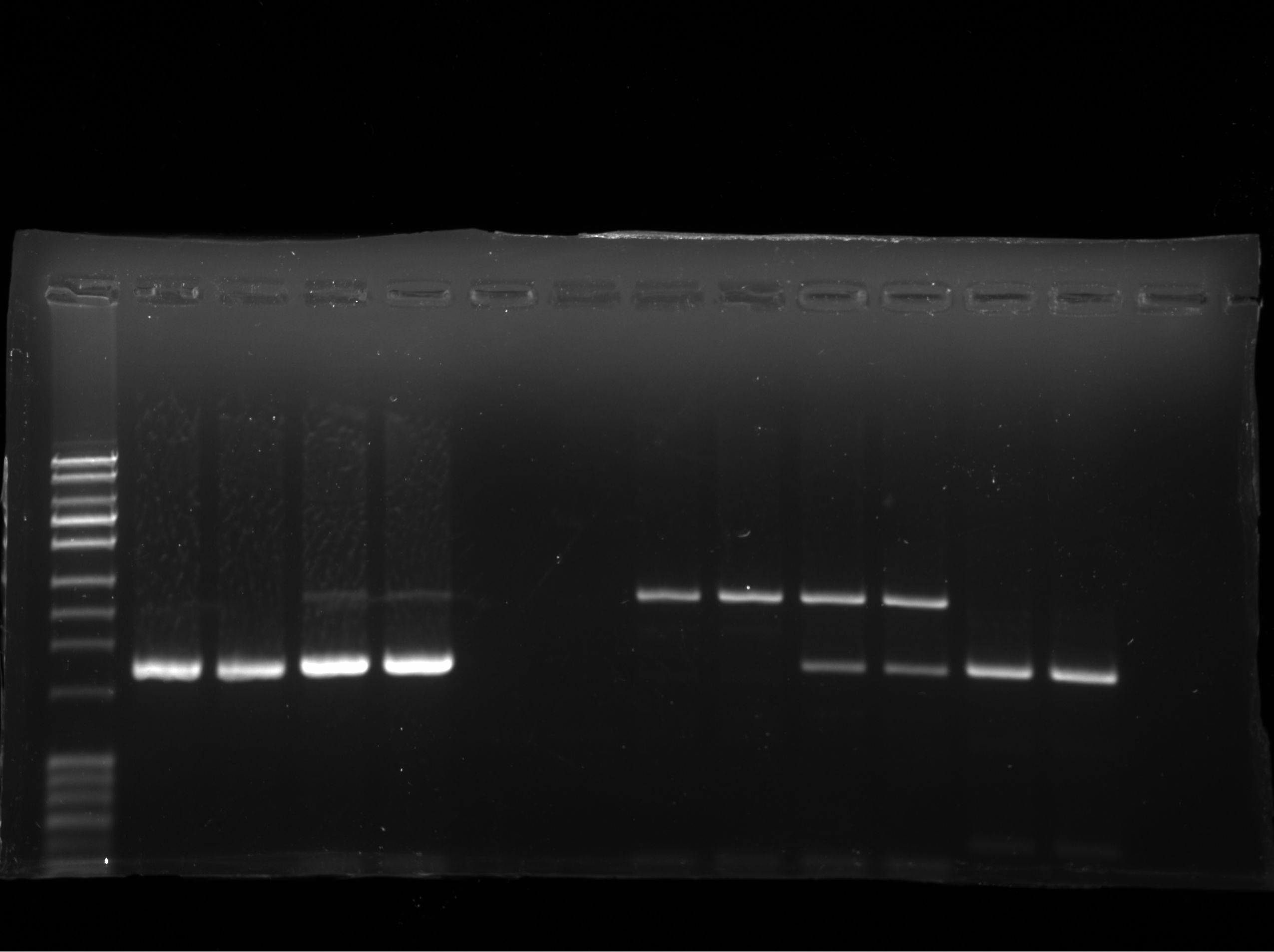

Supplement: Source data 1. [file elife-71279-data1.zip › raw source data images/Figure 5-figure supplement 2-source data 2-panel H_PCR of insertion in rescue line.tif]

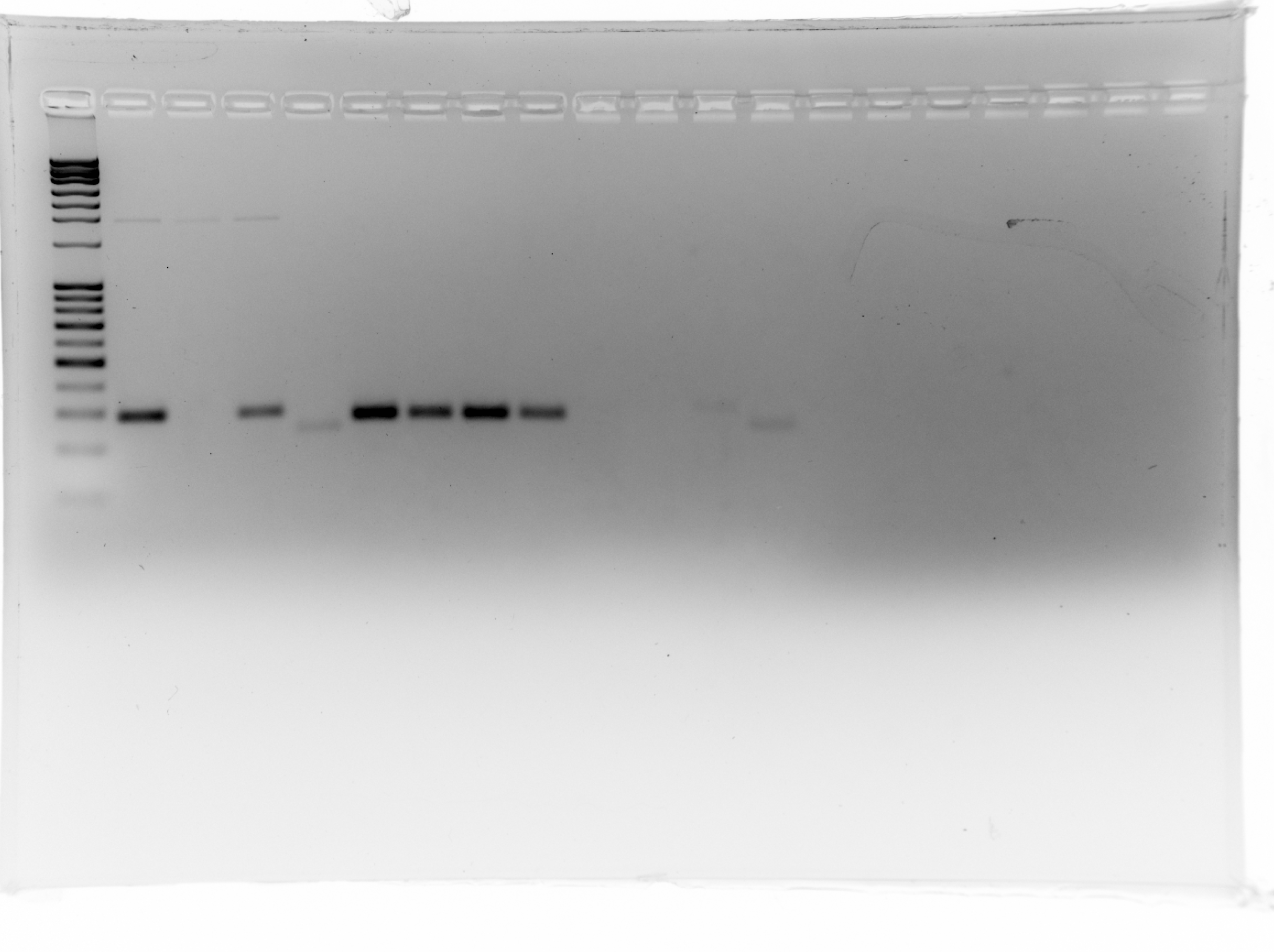

Supplement: Source data 1. [file elife-71279-data1.zip › raw source data images/Figure 5-figure supplement 2-source data 2-panel I_RT PCR of rescue line.tif]

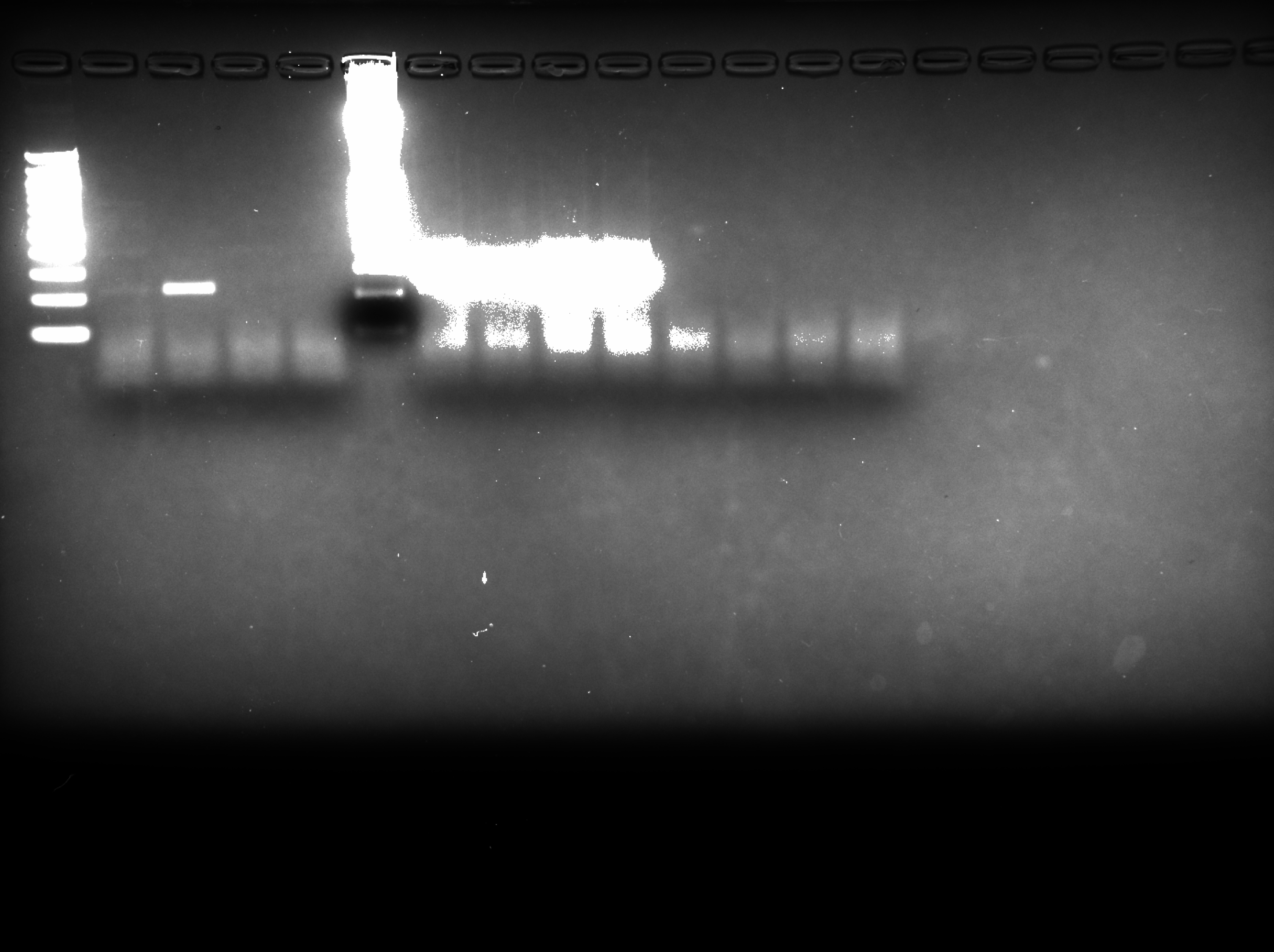

Supplement: Source data 1. [file elife-71279-data1.zip › raw source data images/Figure 7-figure supplement 1-source data 1-panel A_Arp53D expression in WT vs KO_30cycles_longer exposure.tif]

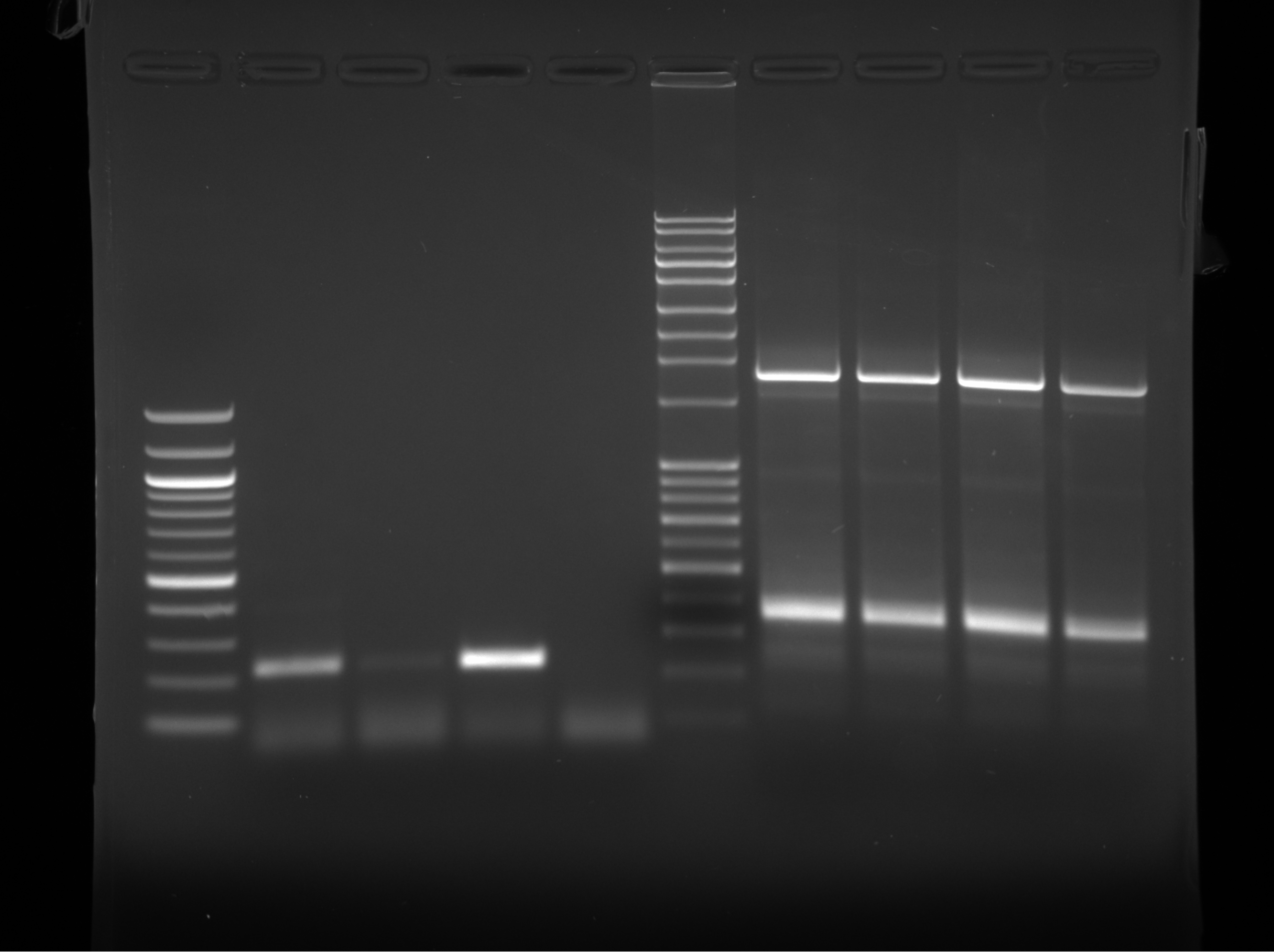

Supplement: Source data 1. [file elife-71279-data1.zip › raw source data images/Figure 7-figure supplement 1-source data 1-panel A_Arp53D RT-PCR_50X cycles.tif]

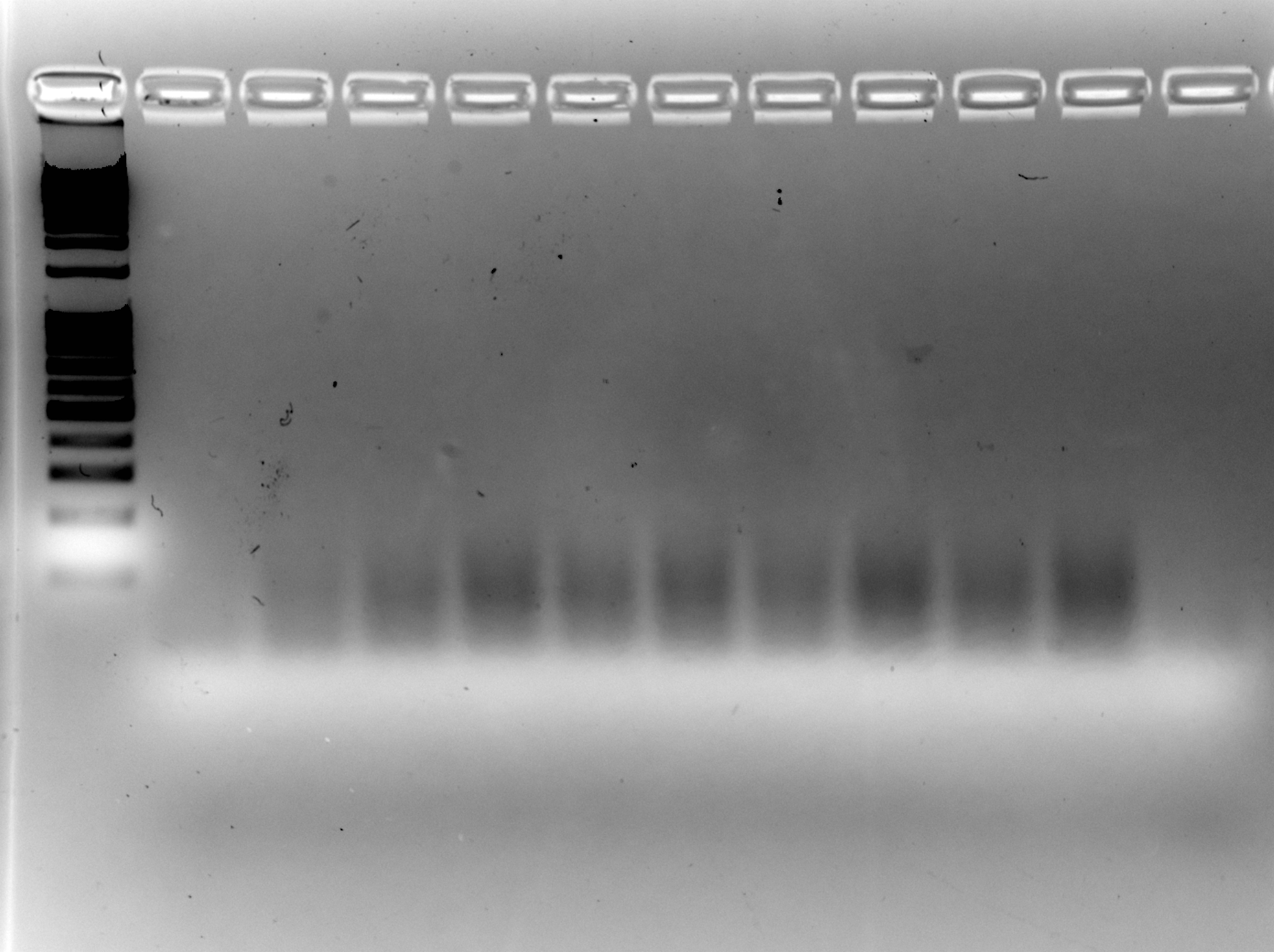

Supplement: Source data 1. [file elife-71279-data1.zip › raw source data images/Figure 7-figure supplement 1-source data 2-panel B_minus RT PCRs.tif]

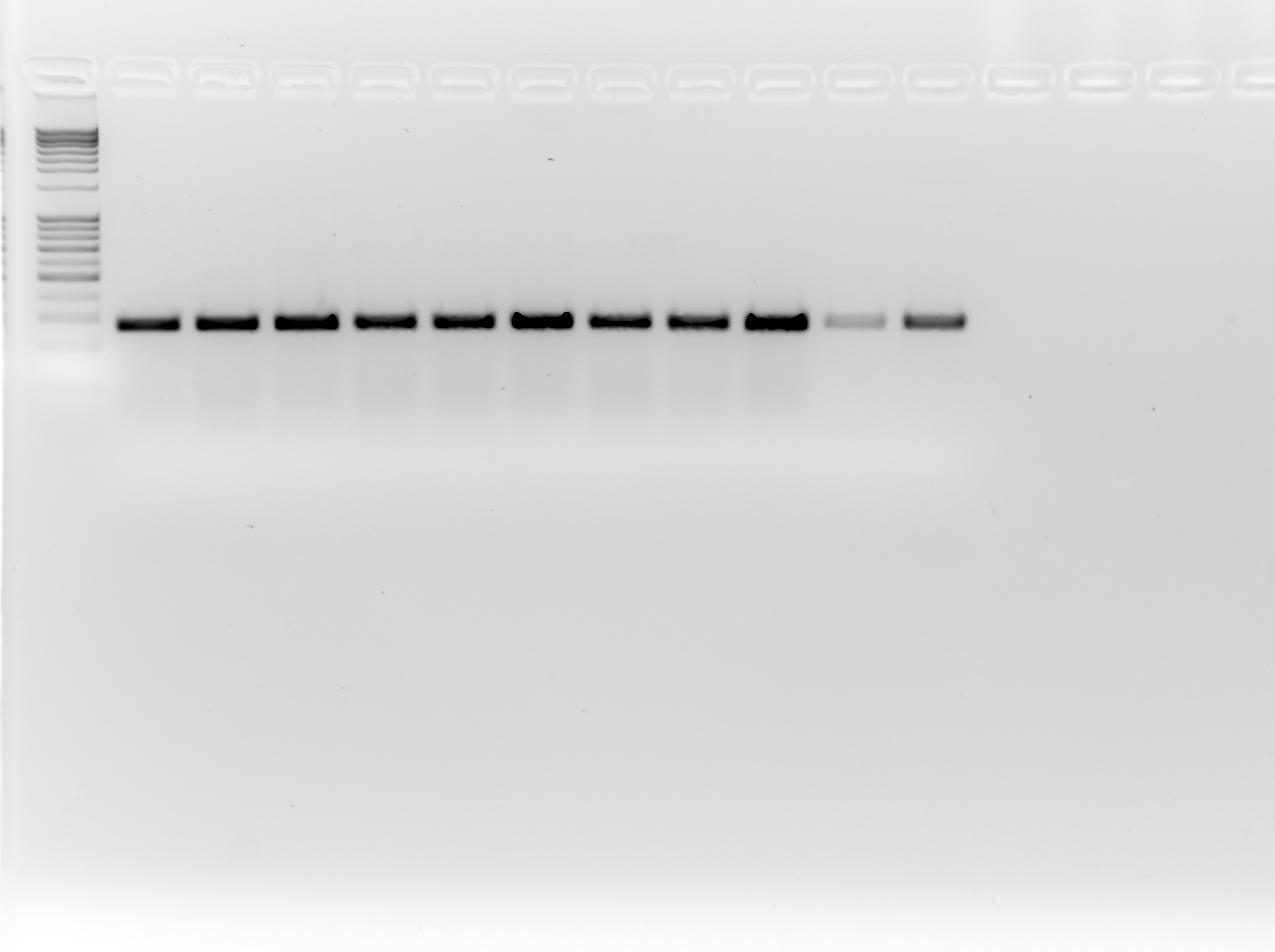

Supplement: Source data 1. [file elife-71279-data1.zip › raw source data images/Figure 7-figure supplement 1-source data 2-panel B_rp49 expression.tif]

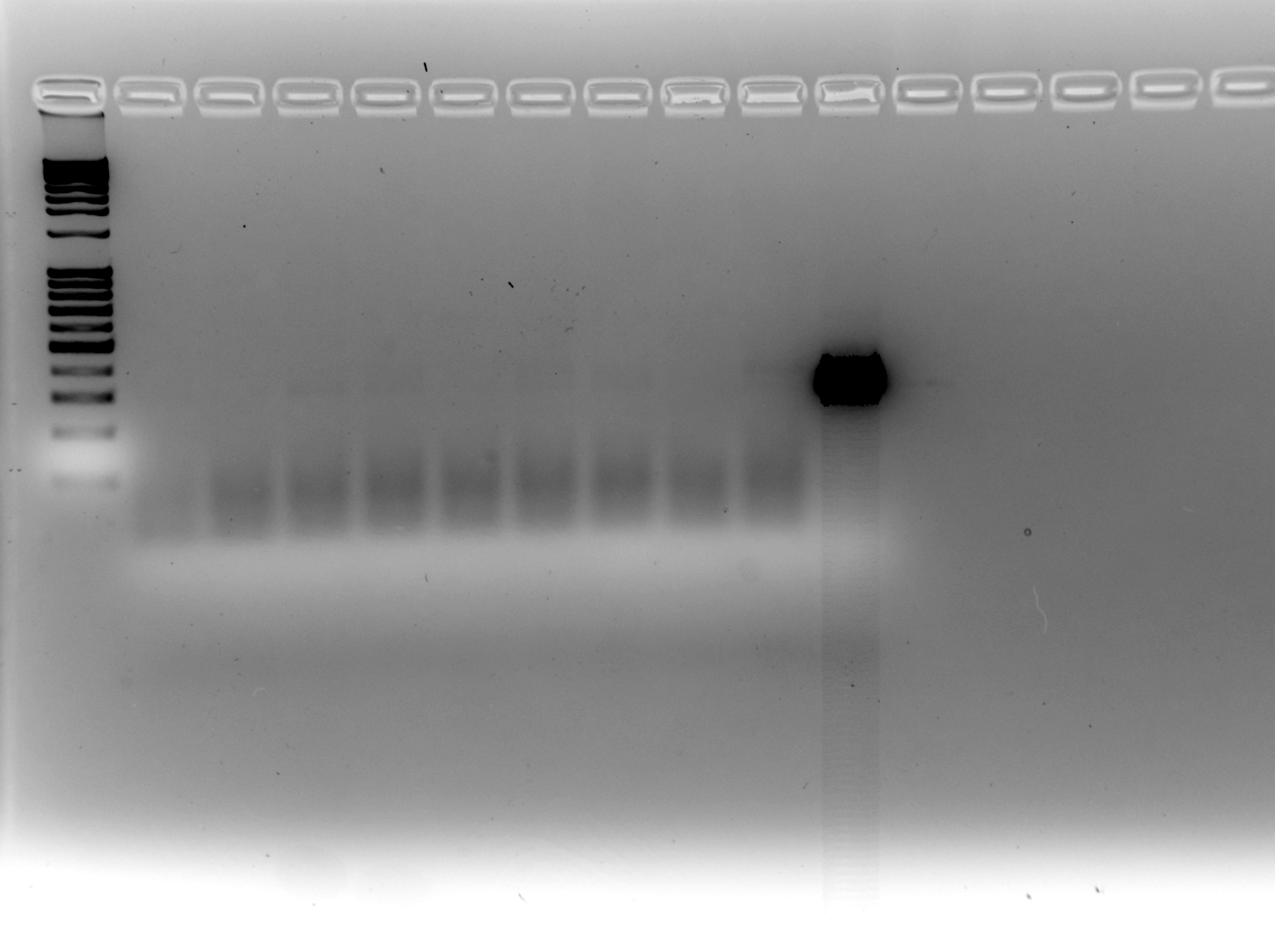

Supplement: Source data 1. [file elife-71279-data1.zip › raw source data images/Figure 7-figure supplement 1-source data 2-panel B_weak Arp53D expression in ovary.tif]
