# Supplementary material for: An actin-related protein that is most highly expressed in Drosophila testes is critical for embryonic development
Source: eLife. 2021 Jul 20;10:e71279. doi: 10.7554/eLife.71279 (PMC8291977; doi:10.7554/eLife.71279)
Supplement: Figure 5—figure supplement 2—source data 1. [file elife-71279-fig5-figsupp2-data1.pdf]

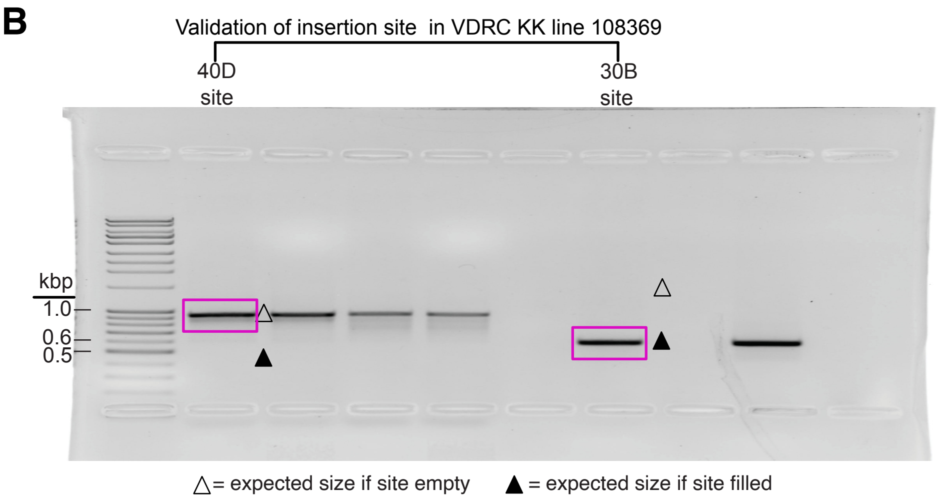

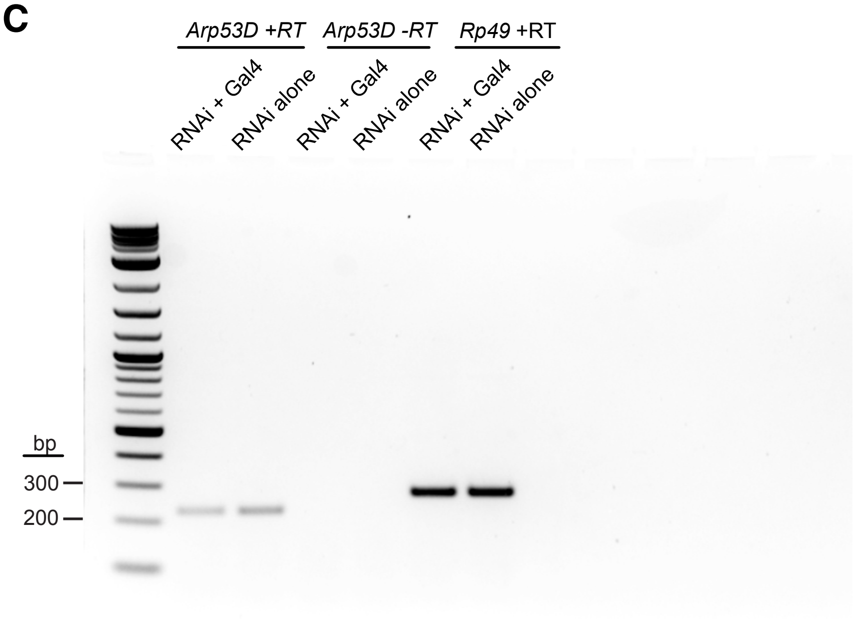

Figure 5-figure supplement 2-source data 1
