# Supplementary material for: An actin-related protein that is most highly expressed in Drosophila testes is critical for embryonic development
Source: eLife. 2021 Jul 20;10:e71279. doi: 10.7554/eLife.71279 (PMC8291977; doi:10.7554/eLife.71279)
Supplement: Transparent reporting form [file elife-71279-transrepform.docx]

***eLife’s* transparent reporting form**

We encourage authors to provide detailed information *within their submission* to facilitate the interpretation and replication of experiments. Authors can upload supporting documentation to indicate the use of appropriate reporting guidelines for health-related research (see [EQUATOR Network](http://www.equator-network.org/%20)), life science research (see the [BioSharing Information Resource](https://biosharing.org/)), or the [ARRIVE guidelines](http://www.plosbiology.org/article/info:doi/10.1371/journal.pbio.1000412) for reporting work involving animal research. Where applicable, authors should refer to any relevant reporting standards documents in this form.

If you have any questions, please consult our Journal Policies and/or contact us: [editorial@elifesciences.org](mailto:editorial@elifesciences.org).

**Sample-size estimation**

- You should state whether an appropriate sample size was computed when the study was being designed
- You should state the statistical method of sample size computation and any required assumptions
- If no explicit power analysis was used, you should describe how you decided what sample (replicate) size (number) to use

Please outline where this information can be found within the submission (e.g., sections or figure legends), or explain why this information doesn’t apply to your submission:

Sample sizes are given in the figure legends and methods. In sum, all fly crosses were conducted with a ratio of 4 females to 1 male with at least 7 total flies per replicate. Flies were allowed to mate and lay eggs for a week and all progeny were counted. For the population cage experiment and embryo collections, generally 100 flies were used as parents.

**Replicates**

- You should report how often each experiment was performed
- You should include a definition of biological versus technical replication
- The data obtained should be provided and sufficient information should be provided to indicate the number of independent biological and/or technical replicates
- If you encountered any outliers, you should describe how these were handled
- Criteria for exclusion/inclusion of data should be clearly stated
- High-throughput sequence data should be uploaded before submission, with a private link for reviewers provided (these are available from both GEO and ArrayExpress)

Please outline where this information can be found within the submission (e.g., sections or figure legends), or explain why this information doesn’t apply to your submission:

For fly crosses, each experiment had a minimum of 5 replicates, and all crosses were conducted a minimum of three times. Graphs that display total progeny counts show the count for each replicate in a given experiment and outliers are included. The population cage experiment was conducted with three replicates. The number of embryos quantified (for nuclear integrity and assessment of fertilization) are provided in the figures and figure legends.

**Statistical reporting**

- Statistical analysis methods should be described and justified
- Raw data should be presented in figures whenever informative to do so (typically when N per group is less than 10)
- For each experiment, you should identify the statistical tests used, exact values of N, definitions of center, methods of multiple test correction, and dispersion and precision measures (e.g., mean, median, SD, SEM, confidence intervals; and, for the major substantive results, a measure of effect size (e.g., Pearson's r, Cohen's d)
- Report exact p-values wherever possible alongside the summary statistics and 95% confidence intervals. These should be reported for all key questions and not only when the p-value is less than 0.05.

Please outline where this information can be found within the submission (e.g., sections or figure legends), or explain why this information doesn’t apply to your submission:

Statistical tests are described in the figure legends and methods. Most p-values were calculated using a t-test except for experiments when percentages were compared between expected and observed, in which case a chi-squared test was conducted (Figure 5 and Figure 8A-B). For the chi-squared tests, calculations and raw data are provided in Supplementary file 1.

(For large datasets, or papers with a very large number of statistical tests, you may upload a single table file with tests, Ns, etc., with reference to sections in the manuscript.)

**Group allocation**

- Indicate how samples were allocated into experimental groups (in the case of clinical studies, please specify allocation to treatment method); if randomization was used, please also state if restricted randomization was applied
- Indicate if masking was used during group allocation, data collection and/or data analysis

Please outline where this information can be found within the submission (e.g., sections or figure legends), or explain why this information doesn’t apply to your submission:

Not applicable.

**Additional data files (“source data”)**

- We encourage you to upload relevant additional data files, such as numerical data that are represented as a graph in a figure, or as a summary table
- Where provided, these should be in the most useful format, and they can be uploaded as “Source data” files linked to a main figure or table
- Include model definition files including the full list of parameters used
- Include code used for data analysis (e.g., R, MatLab)
- Avoid stating that data files are “available upon request”

Please indicate the figures or tables for which source data files have been provided:

All uncropped gel images in the figure supplements are displayed in the source data files and are provided both as labelled figures and as raw, unedited images. The raw data for Figure 5B and Figure 8A-B are given in Supplementary file 1, and the code used to model the population cage experiments is provided on github (<https://github.com/jayoung/Arp53D_popCage>).
